# Differential Processing of Consonance and Dissonance within the Human Superior Temporal Gyrus

**DOI:** 10.3389/fnhum.2016.00154

**Published:** 2016-04-13

**Authors:** Francine Foo, David King-Stephens, Peter Weber, Kenneth Laxer, Josef Parvizi, Robert T. Knight

**Affiliations:** ^1^Biophysics Graduate Group, University of CaliforniaBerkeley, CA, USA; ^2^Helen Wills Neuroscience Institute, University of CaliforniaBerkeley, CA, USA; ^3^Department of Neurology and Neurosurgery, California Pacific Medical CenterSan Francisco, CA, USA; ^4^Stanford Human Intracranial Cognitive Electrophysiology Program, Department of Neurology and Neurological Sciences, Stanford UniversityStanford, CA, USA; ^5^Department of Psychology, University of CaliforniaBerkeley, CA, USA

**Keywords:** electrocorticography (ECoG), consonance and dissonance, auditory cortex, high gamma, music perception

## Abstract

The auditory cortex is well-known to be critical for music perception, including the perception of consonance and dissonance. Studies on the neural correlates of consonance and dissonance perception have largely employed non-invasive electrophysiological and functional imaging techniques in humans as well as neurophysiological recordings in animals, but the fine-grained spatiotemporal dynamics within the human auditory cortex remain unknown. We recorded electrocorticographic (ECoG) signals directly from the lateral surface of either the left or right temporal lobe of eight patients undergoing neurosurgical treatment as they passively listened to highly consonant and highly dissonant musical chords. We assessed ECoG activity in the high gamma (γ_high_, 70–150 Hz) frequency range within the superior temporal gyrus (STG) and observed two types of cortical sites of interest in both hemispheres: one type showed no significant difference in γ_high_ activity between consonant and dissonant chords, and another type showed increased γ_high_ responses to dissonant chords between 75 and 200 ms post-stimulus onset. Furthermore, a subset of these sites exhibited additional sensitivity towards different types of dissonant chords, and a positive correlation between changes in γ_high_ power and the degree of stimulus roughness was observed in both hemispheres. We also observed a distinct spatial organization of cortical sites in the right STG, with dissonant-sensitive sites located anterior to non-sensitive sites. In sum, these findings demonstrate differential processing of consonance and dissonance in bilateral STG with the right hemisphere exhibiting robust and spatially organized sensitivity toward dissonance.

## Introduction

Simultaneous pitch combinations form the building blocks of harmony, a fundamental characteristic of Western tonal music. These pitch relationships can be described as either consonant (often associated with pleasantness) or dissonant (often associated with unpleasantness). While theories relating these pitch combinations to their perceived esthetics have been around since the time of Pythagoras and have enjoyed over two centuries of debate, behavioral evidence indicates that pitch intervals with simple frequency ratios such as an octave (2:1) or a perfect fifth (3:2) tend to be perceived as consonant or pleasant, while intervals with more complex ratios such as a minor second (256:243) or a major seventh (243:128) tend to be perceived as dissonant or unpleasant (Malmberg, 1918; Guernsey, 1928; Plomp and Levelt, 1965; Schellenberg and Trehub, 1994; Schwartz et al., 2003; McDermott et al., 2010). Sensitivity to consonance and dissonance has been observed in infants (Schellenberg and Trehub, 1994; Trainor et al., 2002; Perani et al., 2010; Virtala et al., 2013) as well as in non-musically trained individuals (Koelsch et al., 2000; Regnault et al., 2001; Peretz and Zatorre, 2005; Minati et al., 2009), which suggests that the ability to perceive consonance and dissonance may be an innate and universal aspect of music cognition (Fritz et al., 2009).

The auditory cortex plays a crucial role in multiple aspects of music perception, from basic pitch and rhythm discriminations to complex cognitive tasks in music performance (Peretz and Zatorre, 2005). Not surprisingly, it is also implicated in the perception of consonance and dissonance, as evidenced by lesion studies (Tramo et al., 1990; Peretz et al., 2001; Brattico et al., 2003), non-invasive electrophysiological methods (Tervaniemi et al., 1999; Kuriki et al., 2005; Minati et al., 2009) and functional neuroimaging techniques (Foss et al., 2007; Minati et al., 2009; Daikoku et al., 2012). Multiple findings support a spatial and hierarchical organization for pitch processing within the superior temporal gyrus (STG), where anterolateral regions of the auditory cortex (including lateral Heschl’s gyrus and anterior areas of non-primary auditory cortex) are attuned to more complex pitch stimuli (Wessinger et al., 2001; Patterson et al., 2002; Penagos et al., 2004; Schönwiesner and Zatorre, 2008; Chevillet et al., 2011; Norman-Haignere et al., 2013). As consonance and dissonance are perceptual products of pitch combinations, it remains to be seen whether this spatial organization extends to the processing of consonant and dissonant pitch intervals in anterolateral regions of the auditory cortex.

Intracranial studies in neurosurgical patients provide a rare opportunity to obtain rich electrophysiological signals at a higher temporal resolution compared to fMRI, as well as a higher spatial resolution and a broader range of spectral information compared to scalp EEG. Recent investigations using depth electrodes have shown significant differences between auditory evoked potentials in response to consonant and dissonant chords within primary auditory cortex (Fishman et al., 2001; Dellacherie et al., 2009) as well as in the amygdala, orbitofrontal cortex, and anterior cingulate gyrus (Dellacherie et al., 2009), with theta and alpha band activity in the amygdala causally influencing activity in the orbitofrontal cortex and auditory cortex (Omigie et al., 2015). However, these findings were limited to frequencies up to 70 Hz. Electrocorticographic (ECoG) studies have shown that cortical activity in the high gamma frequency range (γ_high_, >70 Hz) has a high signal-to-noise ratio and is reliable in tracking neuronal activations in various functional modalities, including auditory (Crone et al., 2001a; Edwards et al., 2005; Trautner et al., 2006), language (Crone et al., 2001b; Canolty et al., 2007; Brown et al., 2008; Towle et al., 2008; Flinker et al., 2011), and music (Potes et al., 2012; Sturm et al., 2014) related tasks. Specifically, γ_high_ activity has been shown to track changes in the sound intensity (Potes et al., 2012) and the presence of vocal components in music (Sturm et al., 2014). Additionally, key features in speech sounds have been accurately reconstructed using γ_high_ activations in lateral STG (Pasley et al., 2012). Cortical γ_high_ activity has been linked to neuronal spiking activity, and is believed to emerge from synchronous firing of neuronal populations (Mukamel et al., 2005; Liu and Newsome, 2006; Allen et al., 2007; Belitski et al., 2008; Ray et al., 2008). Thus, we recorded ECoG activity from eight subjects undergoing neurosurgical treatment in order to investigate the spatiotemporal dynamics of cortical γ_high_ activations on the lateral surface of the STG during the perception of highly consonant and highly dissonant musical stimuli. Based on existing animal and human literature, we hypothesized differential γ_high_ activation in response to consonant and dissonant chords in the human STG.

## Materials and Methods

### Subjects

**FIGURE 1:**
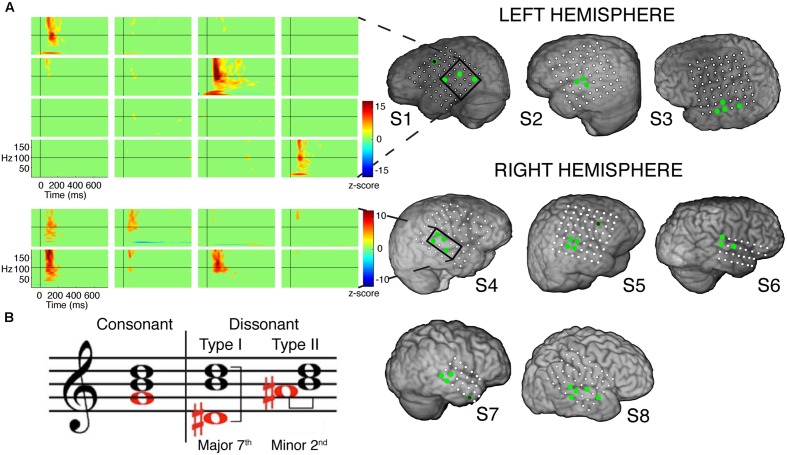
**(A)** Electrode coverage of ECoG subjects; spatiotemporal responses to chord stimuli in subjects S1 and S4. Electrodes with significant activity (*p* < 0.05 after FDR multiple comparison correction, ≥100 ms in duration) in response to chord stimuli are denoted in green. Non-STG electrodes with significant activity (1 each in subjects S1, S5, and S7) are denoted in black with a green border. Event-related spectral perturbations (ERSPs) are shown locked to stimulus onset for the boxed electrodes in subjects S1 (LH coverage) and S4 (RH coverage). Color scale represents statistically significant changes in power compared to a bootstrapped surrogate distribution. **(B)** Chord types used in the experimental design. From L to R: an example of a consonant chord, a dissonant type I chord containing a major seventh interval, and a dissonant type II chord containing a minor second interval.

Eight subjects participated in the study at Stanford Medical Center (*n* = 5) and at California Pacific Medical Center (CPMC; *n* = 3) while undergoing surgical treatment for medically intractable epilepsy. They were implanted with subdural intracranial electrodes spaced one centimeter apart over the left (subjects S1–3) or right lateral temporal and inferior frontal cortices (subjects S4–8) to localize the source of seizures (**Table 1**; **Figure 1A**). All medical treatment including the location of electrode placement was solely determined by the clinical needs of the patient. All subjects gave informed written and oral consent to participate in the study, in accordance with the Declaration of Helsinki. The CPMC research institute and Stanford institutional review boards approved the research that was conducted at each respective location.

**Table 1:** Electrocorticographic (ECoG) subjects.

Subject	Sex	Age	Handedness	Hemisphere coverage	Hospital
S1	M	24	Right	Left	CPMC
S2	F	49	Right	Left	CPMC
S3	F	38	Right	Left	Stanford
S4	M	47	Right	Right	CPMC
S5	M	25	Right	Right	Stanford
S6	M	22	Right	Right	Stanford
S7	M	68	Right	Right	Stanford
S8	F	65	Right	Right	Stanford

*F, Female; M, male; CPMC, California Pacific Medical Center.*

### Task and Stimuli

All subjects participated in a target detection task. Subjects were instructed that they were going to hear musical sounds played on the piano. They were not informed that these sounds were either consonant or dissonant chords, and were therefore naïve to the purpose of this study. They were further instructed that they would sometimes hear a target non-musical sound (a cat’s meow), and they were to count the number of target sounds they heard and report the number at the end of each block of trials. Cortical responses to only the non-target consonant and dissonant chords were considered for analysis.

The consonant and dissonant chords were created in piano timbre using Sibelius 6 software (Avid Technology, Inc.) and digitized using Adobe Soundbooth software with a sampling rate of 44.1 kHz and 16-bit precision. All consonant chords were root position major triads built on each of 12 notes (C_4_ to B_4_), based on the Western classical theory of harmony. Each consonant chord contained a perfect fifth interval between the bottom and top notes. Two dissonant chords were created from each consonant chord by either shifting the bottom note down to form a major seventh interval with the top note (dissonant I), or up to form a minor second interval with the middle note (dissonant II; **Figure 1B**). We used chords in piano timbre as greater activation of bilateral STG has been observed during the perception of harmonic tone complexes in comparison to pure tones (Hall et al., 2002). Additionally, chords in piano timbre have consistently been used in numerous studies involving the perception of consonance and dissonance (Blood et al., 1999; Peretz et al., 2001; Regnault et al., 2001; Minati et al., 2009; Marin et al., 2015), and have been shown to elicit more incremental pleasant/unpleasant ratings than chords in other timbres, such as organ (Blood et al., 1999).

All stimuli were normalized in sound intensity, lasted approximately 700 ms, and were presented with a jittered inter-stimulus interval (ISI) of 1.0 s ± 200 ms (random jitter). Equal numbers of consonant and dissonant chords were presented, and target sounds made up approximately 12.5% of the total stimuli. For subject S1, one block of consonant chords was presented first, followed by one block of dissonant chords. Each block contained 48 stimulus presentations. For subjects S4–S7, both stimuli were presented in pseudo-random order in two blocks. For subjects S2, S3 and S8, four blocks of stimuli were presented in the following order: one block of all consonant chords, one block of all dissonant chords, and two blocks of both stimuli in pseudo-random order. Consequently, subjects S2, S3, and S8 had twice as many trials as the other subjects.

All subjects were presented with the stimuli using EPrime Software (Psychology Software Tools, Inc.) on a Dell Precision M4600 laptop (Dell, Inc.) with two speakers placed in front of them. The speakers were fed directly into the recording system in order to record both stimulus presentation and electrophysiological signals simultaneously.

### Data Acquisition

For subjects at Stanford Medical Center, subdural electrophysiological signals and peripheral auditory channels were acquired using a Tucker Davis Technologies recording system with a 256-channel amplifier and Z-series digital signal processing board. Electrophysiological signals were recorded at a sampling rate of 1526 Hz with a selected subdural electrode as initial reference, and peripheral auditory channels were recorded at a sampling rate of 24.4 kHz. For subjects at CPMC, subdural electrophysiological signals and peripheral auditory channels were acquired using a Nihon Kohden recording system with a 128-channel JE-120A amplifier at a sampling rate of 1 kHz. Electrophysiological signals were recorded with two selected subdural electrodes as reference.

### Data Preprocessing

The raw electrophysiological signals were manually inspected by a neurologist in order to identify and remove pathological channels and epochs of ictal activity that had spread to other non-epileptic channels. Channels with other abnormal signals were also removed. All remaining channels were notch filtered at 60 Hz, band-pass filtered from 1 to 200 Hz and re-referenced to a common averaged reference defined as the mean of all remaining channels. Speaker channels that were recorded simultaneously with electrophysiological activity were manually inspected to mark the onsets and offsets of the stimuli. Trials that overlapped with ictal activity or contained abnormal signals were removed. All analyses were done using custom scripts written in MATLAB (The MathWorks, Inc.).

### Data Analysis

Event related spectral perturbations (ERSPs) were created using a similar method as employed by Flinker et al. (2011). A time-frequency representation of the ECoG signal was constructed by computing its power series for multiple spectral bands defined by using center frequencies logarithmically spaced from 1 to 200 Hz with a fractional bandwidth of 20% of the center frequency. To compute the power series for each spectral band, the ECoG signal was transformed into the frequency domain using an N-point fast Fourier transform (FFT), multiplied with a frequency domain Gaussian filter, and then transformed back into the time domain using an inverse FFT. A Hilbert transform was applied to each signal, and the power estimate was obtained by squaring its absolute value. Event-related power averages (from 0 to 750 ms of stimulus onset) were calculated, baseline corrected (within -200 to 0 ms pre-stimulus onset) and assessed for statistical significance.

Statistical significance was assessed using a similar bootstrapping method as employed by Canolty et al. (2007). A normal distribution of 1000 surrogate ERSPs was created by randomly generating time stamps (equal to the number of actual stimuli onsets) for each ERSP across the entire task, excluding periods of ictal activity or other artifacts. Each time-frequency point in the real ERSP was then expressed using a *z*-score based on the mean and standard deviation of the surrogate distribution of ERSPs. A false discovery rate (FDR) multiple comparisons correction of *q* = 0.05 was applied (Benjamini and Hochberg, 1995).

Cortical sites with ERSPs showing significant activity that extended for either less than 100 ms in duration and/or were not located within the STG were excluded from analysis. Statistical differences between the ERSPs of consonant and dissonant chords were calculated using a non-parametric Wilcoxon rank-sum test with FDR multiple comparisons correction at *q* = 0.05. For each trial, the average raw power value between 75.9 and 144.5 Hz and a fixed temporal duration determined by the onset and offset of significant activity in the ERSP was computed, and the average power values for consonant chord stimuli were tested against those for dissonant chord stimuli. Statistical differences between the ERSPs of dissonant I chords and dissonant II chords were calculated in a similar manner. Statistical differences between averaged ERSPs were calculated by computing the average raw power value between 75.9–144.5 Hz and 75–200 ms post-stimulus onset.

Single-trial γ_high_ traces were plotted by first band-pass filtering the entire ECoG signal from 70 to 150 Hz. Next, event-related epochs were calculated, baseline corrected (within -200 to 0 ms pre-stimulus onset) and expressed as the percent change compared to baseline power.

Spatial relationships between cortical sites exhibiting differential responses to consonant and dissonant chords were assessed by superimposing all electrodes located within the STG for all 8 subjects onto a standardized MNI brain and running a *post hoc* Kruskal–Wallis one-way analysis of variance on the standardized coordinates of significantly activated electrodes in the y- and z-dimensions within each hemisphere.

### Behavioral Study

Ten healthy subjects (six males, four females; mean age: 27.4 years, *SD*: 2.72 years) participated in a separate behavioral study presented using EPrime Software (Psychology Software Tools, Inc.) on a Dell Precision M4600 laptop (Dell, Inc.). The same consonant and dissonant chords as described above were played in pseudo-random order, each lasting approximately 700 ms. After hearing each chord, subjects were instructed to enter a rating between -3 and 3 on the keyboard, representing the range from “very unpleasant” to “very pleasant.” At least four repetitions of each chord were played, and subjects were asked to use the full rating scale. All subjects gave informed written and oral consent to participate in the study in accordance with the Declaration of Helsinki, and the study was approved by the University of California, Berkeley Committee for Protection of Human Subjects.

### Roughness Calculation

To quantify the degree of roughness/sensory dissonance in the stimuli, each chord was analyzed using an algorithm developed by MacCallum and Einbond (2008) in Max/MSP which is based on Parncutt’s dissonance calculation model (Parncutt, 1989; Parncutt and Strasburger, 1994). A roughness value was generated for each chord on a scale from 0 to 7 with increasing values representing increasing degrees of roughness. A Kruskal–Wallis one-way analysis of variance was run on the roughness values, and differences between chord types were assessed using a *post hoc* Wilcoxon rank-sum test with Bonferroni correction for multiple comparisons. Correlation analyses between roughness measures and the mean normalized change in γ_high_ power between 100 and 200 ms post-stimulus onset for each chord were performed using Spearman’s rank correlation.

## Results

A total of 32 electrodes (3–5 electrodes per subject) showed at least 100 ms of significant activity in the high gamma (γ_high_, 70–150 Hz) frequency range compared to baseline in response to the chord stimuli (*p* < 0.05 after FDR multiple comparison correction; **Figure 1A**). Of the 32 electrodes, 29 (91%) were located within the STG. Significant activity was observed as early as 50 ms post-stimulus onset, and varied in temporal duration across electrodes (temporal onsets ranged between 50 and 125 ms post-stimulus onset, while offsets ranged between 175 and 350 ms post-stimulus onset).

**FIGURE 2:**
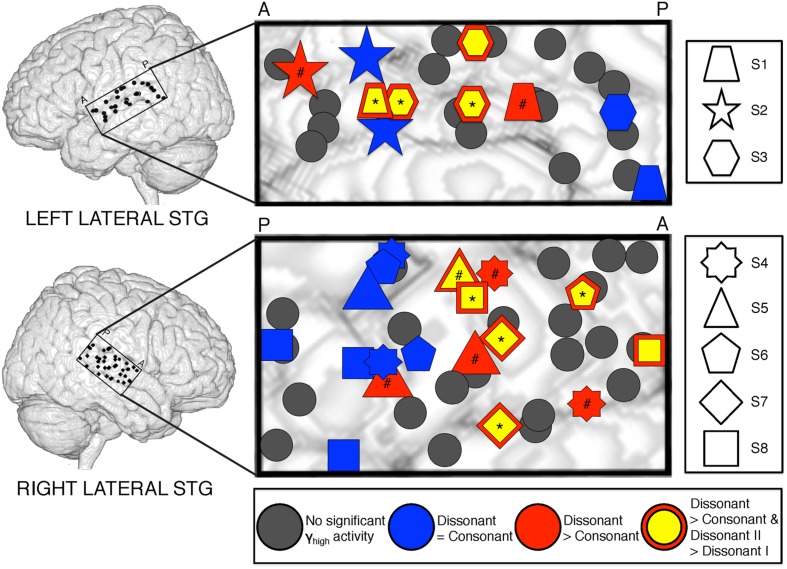
**Spatial distribution of electrodes with differential responses to consonant and dissonant chords in left and right lateral STG.** Electrodes located within the STG for all subjects are shown superimposed on a model brain for each hemisphere. Gray electrodes show minimal or no significant γ_high_ (70–150 Hz) activity in response to any chord type; blue electrodes show no difference in γ_high_ activity between consonant and dissonant chords (Wilcoxon rank-sum, *p* > 0.05); red electrodes show increased γ_high_ activity in response to dissonant chords than consonant chords (Wilcoxon rank-sum, *p* < 0.05); yellow electrodes with a red border show greater γ_high_ activity in response to dissonant type II chords than dissonant type I chords (Wilcoxon rank-sum, *p* < 0.05). Significant electrodes in both Dissonant > Consonant and Dissonant II > Dissonant I conditions after FDR multiple comparisons correction of *q* = 0.05 are marked with a *, and significant electrodes in only the Dissonant > Consonant condition are marked with a #. Onset and duration of FDR-corrected significant increases in γ_high_ activity vary per electrode and is detailed in **Table 2**. Each shape denotes an individual subject. P – posterior, A – anterior.

Analysis of significant STG sites in both hemispheres revealed two response types: one that showed increased γ_high_ activity in response to dissonant chords than consonant chords (electrodes denoted in red; Wilcoxon rank-sum *p* < 0.05 for each electrode), and one that showed no difference in γ_high_ activity between chord types (electrodes denoted in blue; Wilcoxon rank-sum *p* > 0.05 for each electrode; **Figure 2**). Of the 16 electrodes denoted in red, 14 remained significant after FDR correction for multiple comparisons. For subjects S2, S3 and S8, similar cortical responses to consonant and dissonant chords were observed irrespective of the order of stimuli presented. We also observed a clear spatial organization in the right STG, where cortical sites exhibiting greater γ_high_ activity in response to dissonant chords (red) were located anterior to sites with no difference in γ_high_ activity (blue). A significant effect of electrode position in the y-dimension [χ^2^(1) = 8.6, *p* = 0.003] and in the z-dimension [χ^2^(1) = 7.59, *p* = 0.006] of MNI space was observed. This spatial distinction was not observed in the left STG [y-dimension: χ^2^(1) = 0.18, *p* = 0.67; z-dimension: χ^2^(1) = 0.41, *p* = 0.52].

**FIGURE 3:**
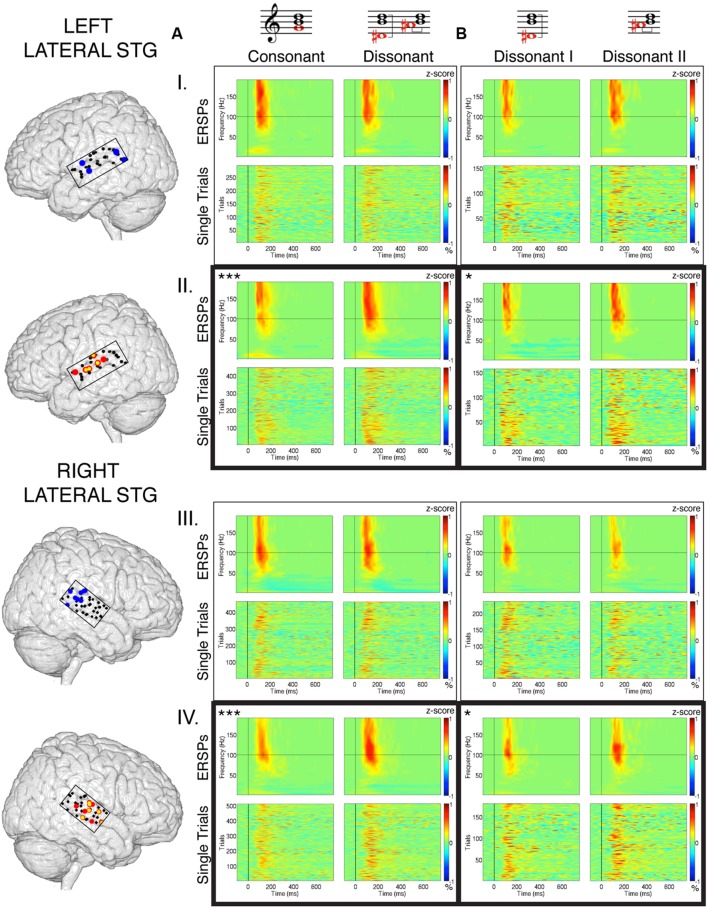
**Averaged ERSPs and Single Trial γ_high_ responses to consonant vs. dissonant chords **(A)** and dissonant I vs. dissonant II chords **(B)** for electrodes denoted in blue (I) and red (II) in the left hemisphere, and electrodes denoted in blue (III) and red (IV) in the right hemisphere.** ERSPs and single trial responses are shown between -100 and 750 ms of stimulus onset. ERSPs are expressed in terms of a *z*-score normalized between -1 and 1 across all subjects. Single trial responses are expressed in terms of the % change in γ_high_ activity compared to baseline activity (-200 to 0 ms pre-stimulus onset) normalized between -1 and 1 across all subjects. Number of electrodes per group: IA, IB, IIB – 4; IIA, IVB – 6; IIIA, IIIB – 8; IVA – 10. Averaged ERSPs showing increased γ_high_ activity between 75 and 200 ms post-stimulus onset for dissonant chords compared to consonant chords as well as for dissonant type II chords compared to dissonant type I chords are boxed. *p < 0.05, ***p < 0.001 (Wilcoxon rank-sum).

**FIGURE 4:**
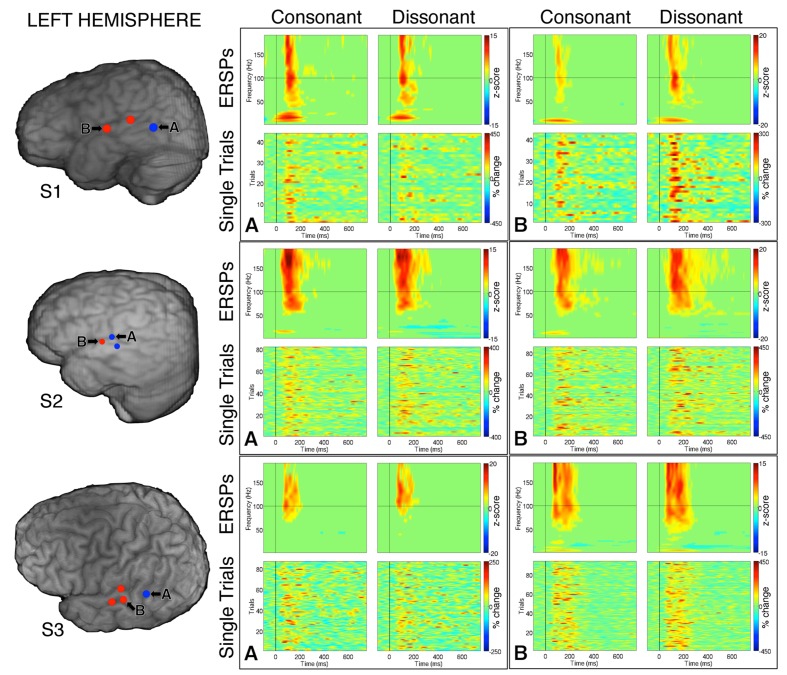
**Event-related spectral perturbations and Single Trial γ_high_ responses to consonant vs. dissonant chords for one example electrode per response type (marked A and B respectively) for each subject with left hemisphere electrode coverage.** All electrodes with significant γ_high_ activity are included in individual subject brain images (left).

**FIGURE 5:**
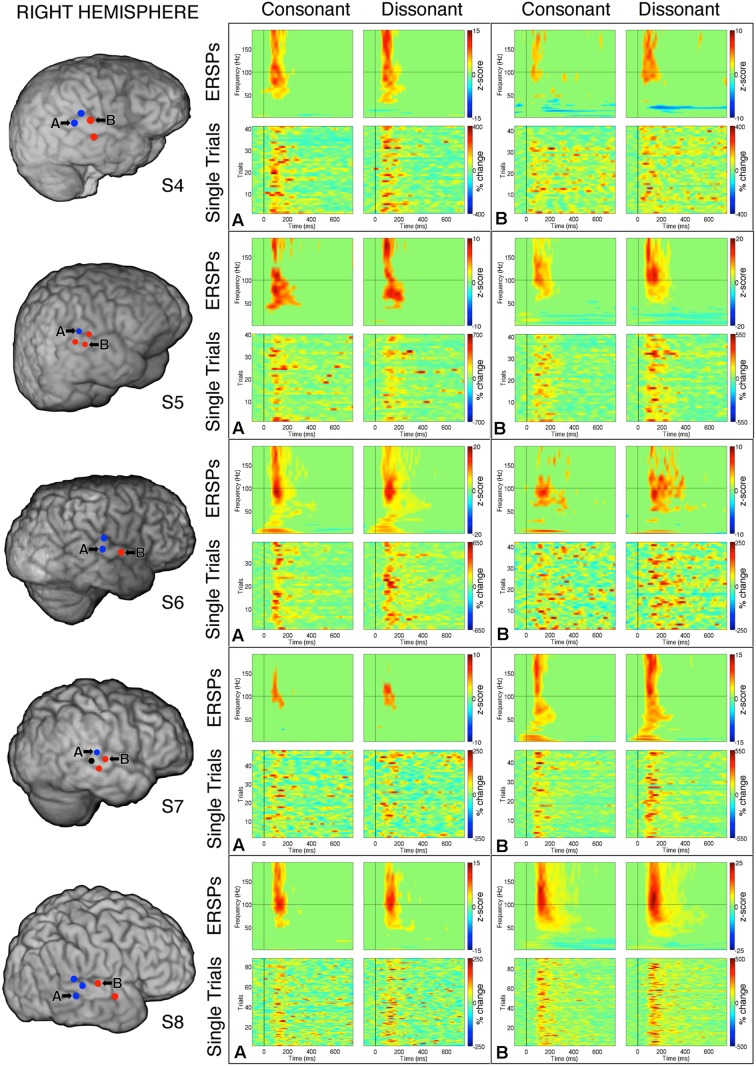
**Event-related spectral perturbations and Single Trial γ_high_ responses to consonant vs. dissonant chords for one example electrode per response type (marked A and B respectively) for each subject with right hemisphere electrode coverage.** All electrodes with significant γ_high_ activity are included in individual subject brain images (left). The electrode marked ‘A’ in subject S7 met the statistical requirement to be categorized in the blue response type group, but was not included in the analysis because significant activation was less than 100 ms.

Within each hemisphere, ERSPs showing the intensity and duration of significant γ_high_ activity were averaged over cortical sites for each response type (LH: red – 6 sites, blue – 4 sites; RH: red – 10 sites, blue – 8 sites; **Figure 3**). In both hemispheres, averaged ERSPs for cortical sites denoted in red showed a significant increase in γ_high_ activity in response to dissonant chords compared to consonant chords between 75 and 200 ms post-stimulus onset (Wilcoxon rank-sum, *p* < 0.001; **Figure 3A**). Averaged ERSPs for cortical sites denoted in blue showed no significant difference between chord types (Wilcoxon rank-sum, *p* > 0.05). Single trial analyses also showed a similar effect, with responses consistently observed across individual trials. (See **Figures 4** and **5** where ERSPs and single trial activity of one example electrode per response type are shown for each subject, and **Table 2** where the duration of significant increase in γ_high_ activity are provided for each electrode denoted in red.) Note that 1 out of the 32 electrodes investigated showed γ_high_ responses that were significantly greater for consonant chords than dissonant chords (**Figure 5**, S7, marked in black).

**Table 2:** Duration of increase in γ_high_ activity for (1) dissonant chords vs. consonant chords and (2) dissonant II chords vs. dissonant I chords for significant STG electrodes denoted in red and marked with a * or # in **Figure 2** (Wilcoxon rank-sum with FDR correction of *q* = 0.05).

Subject	Electrode	Duration of significant γ_high_ activity (ms)	
		Dissonant > Consonant	Dissonant II > Dissonant I
**Left hemisphere**
S1	B	75–225	150–225
	Other	75–200	Not significant
S2	B	150–250	Dissonant I > Dissonant II
S3	B	75–250	100–250
	Other (L)	50–200	130–200
**Right hemisphere**
S4	B	50–175	Not significant
	Other	50–130	Not significant
S5	B	50–250	Not significant
	Other (L)	85–150	Not significant
	Other (R)	75–175	Not significant
S6	B	100–350	125–200
S7	B	75–175	75–175
	Other	75–225	125–175
S8	B	125–225	150–225

*For each subject, electrodes marked ‘B’ in **Figures 4** and **5** are listed first, followed by the other electrodes (ordered from L to R for subjects S3 and S5).*

Averaged ERSPs and single trials locked to the onset of dissonant type I stimuli were contrasted against those locked to the onset of dissonant type II stimuli to assess response sensitivity toward the two types of dissonant chords (**Figure 3B**). In both hemispheres, a subset of cortical sites denoted in red (LH: 4 out of 6 electrodes, 66%; RH: 6 out of 10 electrodes, 60%) showed a statistically significant increase in γ_high_ activity in response to dissonant II chords as compared to dissonant I chords between 75 and 200 ms post-stimulus onset (Wilcoxon rank-sum, *p* < 0.05; see **Table 2** for respective durations of individual electrodes). These electrodes are denoted in yellow with a red border in **Figure 2**. Of these 10 electrodes, 7 remained significant after FDR correction for multiple comparisons. Averaged ERSPs for cortical sites denoted in blue showed no difference between chord types, with single trials exhibiting a similar effect. Note that 1 out of the 16 electrodes denoted in red showed γ_high_ responses that were significantly greater for dissonant I chords than dissonant II chords (**Table 2**, S2).

**FIGURE 6:**
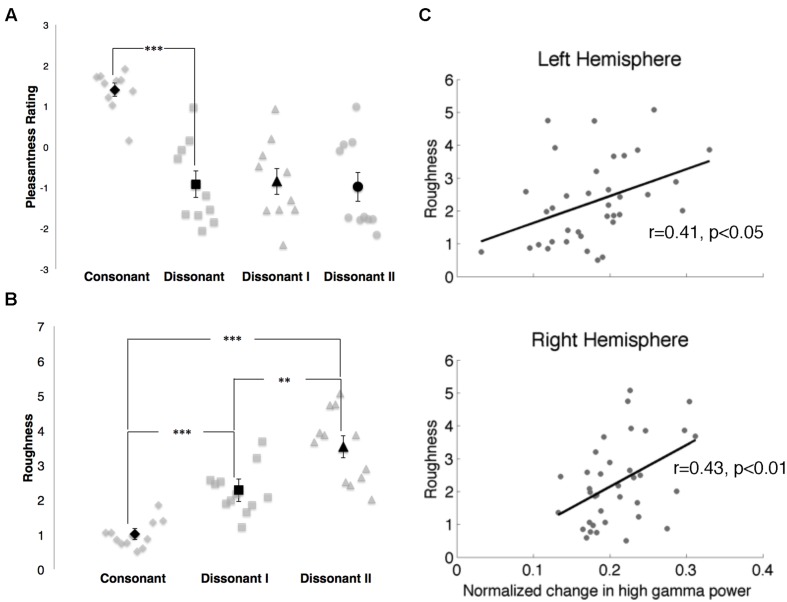
**(A)** Pleasantness ratings for consonant, dissonant, dissonant I and dissonant II chords as indicated by 10 subjects. Points in gray denote individual subject ratings; points in black denote mean subject ratings. Error bars indicate standard error of the mean ratings. ***p < 0.001 (paired *t*-test). **(B)** Roughness measures for consonant, dissonant I and dissonant II chords. Points in gray denote individual chords. Points in black denote mean roughness values. Error bars indicate standard error of the mean roughness value. **p < 0.01, ***p < 0.001 (Wilcoxon rank-sum with Bonferroni correction). **(C)** Correlations between normalized changes in γ_high_ power and stimuli roughness in both hemispheres. Points in gray denote the mean normalized change in γ_high_ power for each chord and its corresponding roughness value.

In the behavioral study, consonant chords were rated as pleasant (average rating of 1.40 ± 0.16) and dissonant chords were rated as unpleasant (average rating of -0.92 ± 0.33), with a significant difference in perceived valence between the two chord types (*p* < 0.001; **Figure 6A**). While dissonant I and dissonant II chords were both rated as unpleasant (-0.85 ± 0.32 and -0.98 ± 0.36 respectively), no significant difference in perceived valence between the two dissonant chord types was observed.

Roughness measures for each stimulus are shown in **Figure 6B**. Significant differences in roughness measures between groups were observed [χ^2^(2) = 26.06, *p* < 0.000003], with both dissonant I and II chords exhibiting a greater degree of roughness than consonant chords (*p* < 0.001, Wilcoxon rank-sum with Bonferroni correction) and dissonant II chords generating a greater degree of roughness than dissonant I chords (*p* < 0.01, Wilcoxon rank-sum with Bonferroni correction). Additionally, changes in γ_high_ power in response to the stimuli were positively correlated with degree of roughness in both hemispheres (RH: *r* = 0.43, *p* < 0.01; LH: *r* = 0.41, *p* < 0.05, Spearman rank correlation; **Figure 6C**).

## Discussion

We recorded ECoG activity directly from the lateral surface of the temporal lobe of eight subjects to investigate the fine-grained spatial and temporal dynamics of consonance and dissonance perception within the STG. In all subjects, we observed cortical sites that were more responsive toward dissonant chords. As cortical γ_high_ power has been shown to correlate with neuronal firing (Ray et al., 2008), our observations are consistent with electrophysiological depth recordings in the auditory cortex of monkeys and humans, where groups of neuronal populations in primary auditory cortex exhibited greater oscillatory phase-locked activity in response to dissonant chords than consonant chords (Fishman et al., 2001).

Fishman et al. (2001) proposed that the increased cortical activity toward dissonant chords reflects a physiological response to the phenomenon of beats or roughness, a sensory property theorized to be associated with dissonance (Helmholtz, 1885/1954). When two simultaneous components of a complex sound have a difference frequency less than the critical bandwidth (10–20% of the center frequency; Zwicker et al., 1957), amplitude fluctuations in the composite waveform envelope are produced which are perceived as either beats or roughness (Plomp and Levelt, 1965; Terhardt, 1974a,b, 1978). The minor second and major seventh^1^ intervals in our dissonant chords satisfy this criterion, while all three intervals (perfect fifth, major third and minor third respectively) in our consonant chords do not. Additionally, the observed increase in γ_high_ activity in response to dissonant chords occurred ~75 ms post-stimulus onset, a timeframe that is traditionally regarded to involve sensory processing of sound features (Picton, 2010). Given that (i) the subjects were not informed of the valenced properties of the musical stimuli, (ii) they were not asked to make a valence judgment on the stimuli, and (iii) their attention was directed toward a target non-musical chord throughout the study, we hypothesize that the increased γ_high_ activity may reflect heightened neuronal firing in response to the beating/roughness that is generated by the minor second and major seventh intervals in our dissonant stimuli.

Furthermore, 60–66% of these dissonant-sensitive cortical sites showed significantly increased γ_high_ activity in response to dissonant II chords containing the minor second interval, consistent with Fishman et al.’s (2001) finding that the peak spectral amplitude of neural activity in both monkeys and humans was higher in response to minor second intervals than major seventh intervals (Fishman et al., 2001). This is notably in contrast with our observation that there was no significant difference in the level of perceived unpleasantness between dissonant I and dissonant II chords. This suggests that γ_high_ activity in response to dissonant chords within the previously identified subset of cortical sites is not strongly modulated by perceived valence. The fact that a minor second interval is ranked higher in comparative roughness than a major seventh interval (Broadhouse, 1881) and that positive correlations were found between changes in γ_high_ power and the degree of roughness of our stimuli further support the notion that γ_high_ activity in response to a given chord may instead be modulated by the acoustical interactions between the component notes that contribute to their roughness. However, as our stimuli were restricted to three chord types due to experimental time limitations in the epilepsy ICU environment, further studies are needed to investigate how cortical γ_high_ power varies with interval type, degree of roughness and perceived valence across a wider spectrum of intervals.

Our findings also showed a distinct spatial relationship between cortical responses in the right STG, where cortical sites that were more responsive to dissonant chords were located anterior to sites that were not specific to chord type. This is consistent with recent literature describing a similar spatial organization for pitch processing in the human auditory cortex. Several fMRI and EEG studies have demonstrated that anterior and lateral regions of the auditory cortex are sensitive to attributes of pitch such as pitch chroma (Warren et al., 2003; Briley et al., 2013) as well as pitch salience and sound complexity (Patterson et al., 2002; Penagos et al., 2004; Schönwiesner and Zatorre, 2008; Norman-Haignere et al., 2013). Since consonance and dissonance are essentially percepts of simultaneous pitch combinations, we postulate that the right auditory cortex is spatially organized for the processing of pitch relationships, with anterior regions exhibiting increased sensitivity toward dissonant intervals.

As electrode coverage in ECoG recordings is typically limited to a single hemisphere per subject, it can be challenging to investigate cognitive effects involving hemispheric asymmetries. In our study, spatial organization was evaluated within each hemisphere by superimposing all electrodes displaying significant γ_high_ activity across subjects onto a standardized MNI brain and localizing them using a common coordinate reference. We observed that the spatial distinction between cortical sites exhibiting differential responses to consonant and dissonant chords was significant in the right hemisphere but not in the left. While this observation is consistent with multiple studies on brain networks involving music perception that show a dominance of the right over the left hemisphere (Peretz and Zatorre, 2005), it is possible that the lack of spatial organization in the left hemisphere may be due to inter-subject differences in regional cytoarchitecture, as well as the limited number of patients presented in this study with electrode coverage in the left hemisphere (*n* = 3). Furthermore, cortical responses with significant γ_high_ activity were limited to 3–5 sites per subject at an inter-electrode spacing of 1 cm. Since differences in functional responses between phoneme and word stimuli have been reported within 4 mm of cortex (Flinker et al., 2011), it would be interesting to compare our current observations with a finer-grained spatial map of cortical responses sampled at a sub-centimeter resolution within the STG.

## Conclusion

Our study provides evidence for differential processing of consonance and dissonance within bilateral STG. Cortical responses were spatially organized in the right hemisphere, with regions exhibiting increased sensitivity toward dissonance located anterior to non-sensitive regions. These findings demonstrate the ability of ECoG to track fundamental aspects of music perception with high spatial and temporal precision, and provide a platform technology for future studies involving higher-level aspects of music cognition.

## Author Contributions

FF planned and designed research. DK-S, PW, KL, and JP performed the experiments. FF analyzed data. FF and RK interpreted results of experiments. FF prepared figures and drafted manuscript. FF and RK edited and revised manuscript. RK approved final version of manuscript.

## Conflict of Interest Statement

The authors declare that the research was conducted in the absence of any commercial or financial relationships that could be construed as a potential conflict of interest.

## Footnotes

1 In a complex-tone major seventh interval, the difference frequency between the interval’s fundamental tones is larger than the critical bandwidth. However, the second harmonic of the bottom note forms a minor second interval with the first harmonic/fundamental frequency of the top note. The difference frequency between these two harmonics is smaller than the critical bandwidth, thereby contributing to the interval’s perceived roughness.
